# Cavitation Erosion of Protective Coating Based on Cordierite Filler and Epoxy Matrix

**DOI:** 10.3390/ma18051034

**Published:** 2025-02-26

**Authors:** Marko Pavlović, Marina Dojčinović, Jasmina Nikolić, Stanko Aleksić, Nedeljko Tucović, Zoran Čeganjac, Saša Drmanić

**Affiliations:** 1 Innovation Centre of Faculty of Mechanical Engineering, University of Belgrade, 11000 Belgrade, Serbia; 2 Faculty of Technology and Metallurgy, University of Belgrade, 11000 Belgrade, Serbia; rina@tmf.bg.ac.rs (M.D.); jasminanik313@gmail.com (J.N.); drmana@tmf.bg.ac.rs (S.D.); 3 Laboratory of Physics and Chemistry, Institute of Nuclear Science INS Vinca, 11000 Belgrade, Serbia; frakulah@gmail.com; 4 Jugoinspect A.D., 11000 Belgrade, Serbia; ntucovic@gmail.com; 5 Department in Arandelovac, Academy of Professional Studies Sumadija, 34300 Arandelovac, Serbia; zoocega@yahoo.com

**Keywords:** cordierite filler, metal substrate, cavitation resistance, microstructure

## Abstract

The goal of this study is to investigate the surface morphology changes induced by the cavitation erosion of a coating based on cordierite with an epoxy matrix for an aluminum substrate. The literature review shows a certain lack of knowledge regarding the coating’s resistance to wearing induced by water flow, which is a highly important property of the material immersed in or in contact with water streams. The main idea behind the investigation is that such a protective coating will also improve the cavitation erosion resistance of metal substrates. The protective coatings were based on cordierite filler (88 wt.%) and epoxy resin (7 wt.%). The filler, made of a mixture of kaolin, alumina, and talc, is obtained by a sintering procedure that took place at 1350 °C. X-ray diffraction analysis and scanning electron microscopy were employed in the characterization of the produced filler. The adherence of the obtained epoxy-based protective coating and resistance to water flow were tested by the ultrasonic vibration method (i.e., cavitation erosion testing). Scanning electron microscopy was used for analysis of the coating’s morphology upon cavitation erosion. Based on the value of the cavitation erosion rate and the analyzed final surface damage, it was assessed that the investigated protective coating is resistant to cavitation erosion.

## 1. Introduction

The resistance of substrates to various types of erosion (e.g., carbonation, chloride ion erosion, cavitation erosion via water) can be significantly increased by applying epoxy-matrix-based coatings to metal and non-metal surfaces [1,2,3]. Numerous investigations have shown that the high density and stable chemical composition of inorganic film-forming coatings are the most important factors for creating a protective layer on the surface of the material. This layer prevents the corrosive media from the environment (water, moisture, CO_2_, Cl^−^), from being in direct contact with metal elements or concrete in a structure [4,5,6,7,8,9,10]. In order to guarantee the long-term service performance of either industrial or residential metal and/or composite structures, protective coatings have become important as an adjunct technology [10,11,12]. Even though traditional coatings perform exceptionally well in terms of protection, the majority of them are produced with organic solvents and contain a number of hazardous volatile organic compounds (xylene and toluene), which not only harm the environment but also put human health at serious risk [11]. The fundamental objective is to create a protective coating with a low organic content and a high percentage of inorganic filler with a high hardness value and adequate grain size and grain shape distribution to achieve good adherence to the metal substrate in a thin layer. The obtained coating has to be non-toxic, highly erosion-resistant, and easy to clean. According to the research provided by other authors [13,14], currently, the majority of applications for these coatings are found in the metal structural components of buildings, equipment parts that come into direct contact with water, cars, boats, etc.

The performance of predominantly inorganic coatings has increased over time, and their potential for use in engineering practice has grown, owing primarily to the development of micron- and sub-micron-sized materials. Specifically, materials (fillers for coatings) with different grain-size and grain-shape distributions, including very small particles (micron or sub-micron grains), show advantages in terms of adherence to the substrate and evenness of the protective layer. Research has so far shown that adding raw materials such as graphene oxide, nano-silica, nano-Fe_2_O_3_, nano-TiO_2_, and nano-clay may effectively raise the coating density, eliminate microscopic flaws in the coating, and improve the coating’s mechanical characteristics and wear resistance [15,16,17,18,19,20,21,22,23,24]. All of these factors strengthen the metal substrate’s anti-erosion properties. In research [25,26,27,28,29], a large number of different fillers for coatings are included (fillers based on mullite, zirconium silicate, talc, pyrophyllite, cordierite, basalt). Their preparation and application in the composition of protective coatings and coatings for use in foundry was investigated. For the potential application of fillers, filler properties such as strength and hardness, preparation of fillers by grinding and micronization processes, and the size and shape of filler grains play a significant role. These properties of the filler are important to obtain continuous layers of the coating on the substrate, all of which will increase the resistance to wear and cavitation erosion [27,28,29].

A particular kind of wear, known as cavitation, involves the emergence, development, and implosion of vapor in a water flow. Microjets, a cavitation vortex, and intense shock waves induce high temperatures and pressure, which can severely damage the substrate material [21,22,24]. The layers on the material’s surface gradually harden as a result of the energy build up. Thereby, the material progressively loses its ability to deform further, becomes more brittle, and develops fractures that deepen over time as a result of particles being removed from the fracture surface and the crack’s margins, and also of the risk of smaller or bigger pits (holes) [4,23]. If surface damage expands, indicating cavitation-related degradation, the material may be destroyed. As a result, determining and assessing filler grain sizes and grain shapes, in order to optimize them, can be critical for producing protective coatings with a low risk of cavitation erosion.

The goal of this work is to employ micron and sub-micron sized synthetized inorganic fillers with high hardness in a non-toxic protective coating, which can be used as an erosion shield on metal surfaces. A filler based on cordierite was chosen for the research. Raw mineral materials—alumina, talc, and kaolin—were used for the synthesis of fillers based on cordierite.

The laboratory-scale synthesis of cordierite filler-based alumina, kaolin, and talc was performed in order to produce as much of the cordierite phase in the coating as possible. Thus, the obtained properly dispersed micron and sub-micron particles immersed in an epoxy matrix would provide a non-toxic protective covering that might be used as an erosion “shield” for metal (aluminum) substrates. The next step was to systematically investigate the surface morphological changes generated by cavitation erosion on the resulting coating affixed to an aluminum plate. The aluminum substrate was proven to be non-resistant or low-resistant to cavitation erosion [24]. The literature review reveals a significant level of a lack of information about coatings’ resistance to wear caused by water flow, which is an important property of any material submerged in or in contact with water streams.

## 2. Materials and Methods

### 2.1. Raw Materials and Synthesis of the Cordierite Filler

Cordierite (2MgO·2Al_2_O_3_·5SiO_2_), as a filler, offers various advantages regarding the properties needed for a protective coating component. This filler has a low coefficient of thermal expansion, good resistance to thermal shock, good physico-mechanical properties such as hardness, flexural and compressive strength, high refractoriness, inertness towards liquid metal, and good corrosion resistance [19]. For instance, the remarkable resistance of cordierite to heat shock is a result of its extremely low coefficient of thermal expansion. Cordierite crystallizes at approximately 830 °C in an orthorhombic, pseudohexagonal system to μ-cordierite, and has a comparatively low melting point of 1470 °C [20]. The cordierite crystallization causes a shrinkage of about 4.7% [20]. The thermal expansion coefficient of cordierite coatings is 3.21 × 10^−6^ K^−1^ within the 25–400 °C temperature range [20]. Also, in the study conducted by C. Semmler et al. [20], only cordierite coatings could be applied without cracks. The cordierite coatings achieved the lowest thermal conductivity in the 50–200 °C temperature range, with values less than 1.2 W/mK [20].

The filler for the protective coating was made experimentally using the following basic raw materials: kaolin (distributor: Jugo Kaolin D.o.o., Beograd, Serbia), aluminum (III) oxide/alumina (distributor: “Alumina” d.o.o. Zvornik, Bosnia and Hercegovina), and talc (distributor: Credicom International, Beograd, Serbia).

The chemical composition of the raw materials was determined by means of atomic absorption spectrometry on a Perkin Elmer Analyst 300 Instrument. The wavelength range of the device is 185–900 nm. The AAS instrument has an optical dual beam monochromator with 1800 lines/mm, a photo multiplicator detector, and a carrier with 6 lamps with automatic positioning. Loss on ignition values, measured upon firing at 1000 °C, for commercial kaolin, alumina, and talc, were 7.95%, 3.17%, and 6.91%, respectively.

The chemical compositions of the raw components were as follows:

(1) Kaolin—SiO_2_ = 53.87%, Al_2_O_3_ = 28.24%, Fe_2_O_3_ = 1.48%, CaO = 0.64%, Na_2_O = 0.03%, and K_2_O = 0.05%;
(2) Alumina—SiO_2_ = 0.15%, Al_2_O_3_ = 95.12%, MgO = 0.01%, Fe_2_O_3_ = 0.1%, CaO = 0.17%, Na_2_O = 0.04%, and K_2_O = 0.02%;
(3) Talc—SiO_2_ = 60.97%, Al_2_O_3_ = 1.68%, MgO = 28.91%, Fe_2_O_3_ = 2.23%, CaO = 2.95%, Na_2_O = 0.87%, and K_2_O = 0.91%.

The filler mix design included 29 wt.% kaolin, 35 wt.% alumina, and 36 wt.% talc. The raw materials were mixed in the given ratio in an ultra-centrifugal mill (Retsch ZM-1) for 60 min. Subsequently, the homogenized mixture was shaped into tablets using a conventional laboratory hydraulic press (the applied load on the machine was 1 MPa). The sintering was conducted in a Carbolite laboratory chamber furnace. The heating temperature increased from ambient 20° to 1350 °C. An oxidizing atmosphere was used. The following heating regime was used: a continuous heating rate of 10 °C/min up to 1000 °C, followed by a constant heating rate of 5 °C/min between 1000 °C and 1350 °C. A three-hour delay was used at the maximum temperature. The synthetized tablets were crushed upon cooling down and subsequently ground in the ultra-centrifugal mill for additional 60 min.

### 2.2. Instrumental Methods for Characterization of the Synthetized Cordierite Filler

In order to determine the mineral phase composition of the synthesized filler, the X-ray diffraction (XRD) technique was applied on crushed and subsequently pulverized sintered tablets. A Philips X-ray diffractometer, model PW-1710, equipped with a scintillation counter and a curved graphite monochromator, was used. The intensities of the diffracted CuKα X-ray radiation (λ = 1.54178 Å) were measured at room temperature in the intervals of 0.02° *2θ* and 1 *s* in the range from 4 to 65° *2θ*. The X-ray tube was loaded with a voltage of 40 kV and a current of 30 mA, while the slits for directing the primary and diffracted beams were 1° and 0.1 mm.

The microstructural analysis was conducted via a scanning electron microscope (SEM)—JEOL JSM-6610LV (Tokyo, Japan) on the pulverized sintered samples. The powdery samples were coated with the carbon using table-top sputter coater LEICA SCD005 (Wetzlar, Germany). The magnification of the instrument is 5 to 300,000 times. The electron source is a W wire, LaB 6. The voltage is 0.3–30 kV. The instrument works with a vacuum system.

The qualitative mineralogical analysis of the samples was performed under a polarized transmitted light microscope of the brand Jenapol, Carl Zeiss-Jena, Germany. The immersion method (xylene immersion) was used for the qualitative identification of the minerals present. The magnification of the objective is 10 to 50×. The measurement of grain size and the abundance factor was performed on 3500–4000 grains. The analysis of the grain shape and size was performed using the OZARIA 2.5 software package (interval from 0 to 1), where the shape factor was determined as follows: for 0—the cross section corresponds to the shape of a needle, for 1—the cross section corresponds to a circle, while the grain size is given in micrometers (µm). The division according to the grain shape factor was as follows: from 0.0–0.2—angular; from 0.2–0.4—subglobose; from 0.4–0.6—subrounded; from 0.6–0.8 rounded; and from 0.8–1.0—well-rounded grain shape. The photomicrography system Studio PCTV (Pinnacle Systems, Mountain View, CA, USA) was used for recording [30].

### 2.3. Preparation and Mix-Design of the Protective Coating

The established mix-design of the experimental protective coating was as follows: the synthesized filler (88 wt.%), the epoxy resin used as coupling agent (7 wt.%), and CoatOSil™ MP 200 (Manufacturer: Grolman Group, Neuss, Germany) used as the additive (1.2 wt.%). An epoxy functional silane oligomer called CoatOSilTM MP 200 is useful in urethane, epoxy, acrylic, and polysulfide-based coatings as a crosslinker and adhesion booster. It works well with solvent-borne and water-based coatings, where it can aid in strengthening the crosslinking density to enhance adhesion to the substrate, hardness, and chemical resistance. Water (3.8 wt.%) was used as a solvent. Water causes the epoxy matrix to plasticize, a physically based and reversible breakdown process that increases the mobility of macromolecular chains. The components were gradually added with continual mixing in a laboratory mixer. The acquired viscosity of the coating was 1200 cPs, and it was determined by a laboratory viscometer, where a spindle is rotated in the epoxy fluid, according to the ASTM D-2393 standard [31]. The fluid’s resistance to flow is accurately measured in centipoise (cPs).

The coatings were applied in two layers with a brush on a metal (aluminum) plate for testing, and then dried in the air for 60 min. The application procedure of the protective coating was performed accordance to past studies [32,33].

### 2.4. Instrumental Methods for Characterization of the Protective Coating

The standard test method for cavitation erosion according to ASTM G32-16 [34] was employed on the experimentally prepared protective coating. Since the tested material is brittle, the ultrasonic vibratory cavitation method with a stationary sample was used [35].

The sample holder was fixed to the bottom of the water bath. The mechanical vibratory concentrator was immersed in a water bath. The water temperature was maintained constant at 25 ± 1 °C. A 0.5 mm gap separated the sample from the front surface of the vibratory concentrator. Mechanical vibrations at a frequency of 20.0 ± 0.2 kHz were used. The amplitude of mechanical vibrations at the top of the concentrator was 50 ± 2 μm. The gap between the test sample and concentrator was 0.5 mm. Under the front surface of the concentrator and the stationary-tested sample, a substantial cavitation zone was formed. The water bath was cooling the sample to keep it at a constant temperature. A pressure field was created by a continuous water flow, which induced the implosion of the cavitation bubbles on the surface of the sample. The water flow rate was 5–10 mL/s, and the temperature in the bathroom was 25 ± 1 °C. The cavitation periods employed were as follows: 0, 15, 30, 45, and 60 min. Only one sample was tested for all planed exposure times. During each test, the sample was dried, and the mass loss was measured with an analytical precision of ±0.1 mg. The test output represents an average of a minimum of three tests per sample. The cavitation damage results are presented as the mass loss diagram by charting the mass loss values on the ordinate and the time of material exposure on the abscissa. The rate of cavitation erosion was obtained using the least squares method (the tangent of the slope depicts the loss of mass during the period of cavitation activity). The measurements were conducted in accordance with ASTM G32-16. For each set of the tested samples, three samples were used, and the findings represent the mean value of these measurements for each test interval.

The rate of cavitation degradation has no set limits provided in the standard. Both visual inspections and mass loss can be used to detect degradation (supported by microscopic procedures). Real-scale and laboratory-scale models differ from one another as well. In other words, the water force generated in the lab model is practically greater than the force generated on the structural element on a real-sized scale (depending on the size of the tested sample). As a result, testing has been conducted across shorter time periods (e.g., intervals from 15 to 60 min). The cavitation erosion would take a lot longer to develop in a real environment.

The morphology of the samples’ damaged surfaces was examined using a scanning electron microscope (JEOL JSM-6610LV) (method and instrument specified in the preceding sub-section).

## 3. Results and Discussion

### 3.1. Properties of the Synthetized Cordierite Filler

**Figure 1:**
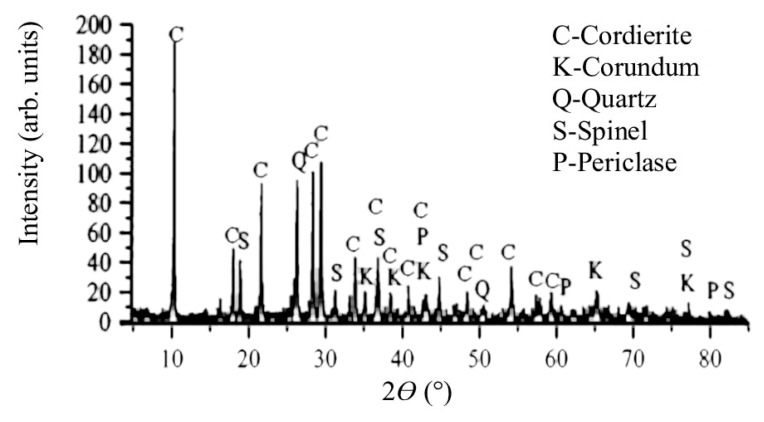
X-ray diffractogram of synthesized cordierite filler.

The major oxides identified in the synthesized filler are as follows: SiO_2_ = 39.91%; Al_2_O_3_ = 44.05%; MgO = 11.35%; Fe_2_O_3_ = 1.55%; and CaO = 1.6%. The LoI measured at 1000 °C is 0.63%. The obtained chemical composition varies from a ‘theoretical’ chemical composition of cordierite [36,37,38]: SiO_2_ ≈ 48%; Al_2_O_3_ ≈ 33%; MgO ≈ 12%; and Fe_2_O_3_ ≈ 2.4%. The conducted synthesis resulted in cordierite and different mineral phases such as corundum, quartz, spinel, and periclase, which can be seen from the X-ray diffractogram in Figure 1.

It can be seen from the diffractogram that the most intense peak of cordierite is at 10°. According to JCPDS 13-294 (International center for diffraction data (https://www.icdd.com/)), it can be seen that there are peaks at 18°, 19°, 21.5°; 26°, 28°, 29° etc., with the peaks between 25 and 30° being only slightly less intense than the peak at 10°. The quartz (JCPDS 46-1045) reflections were present at ~26°, where the most intense peak is for quartz. The most intense peak for spinel is at ~37°, and smaller peaks are at 19°, 32°, 45°, 70°, 77°, and 84° (JCPDS 77-1193). Corundum (JCPDS 46-1212) reflections are observed at 35°, 37°, 44°, 65°, and 77°. The peaks for periclase are present at 44°, 61°, and 80° (JCPDS 82-0512). The Mohs hardness of the observed mineral phases is as follows: cordierite (7–7.5), corundum (9), spinel (8), quartz (7), and periclase (5.5) [39]. The obtained mineral phase blend indicates a crystalline material with high hardness, which is a physical property that is important for a filler used in protective coatings.

**Figure 2:**
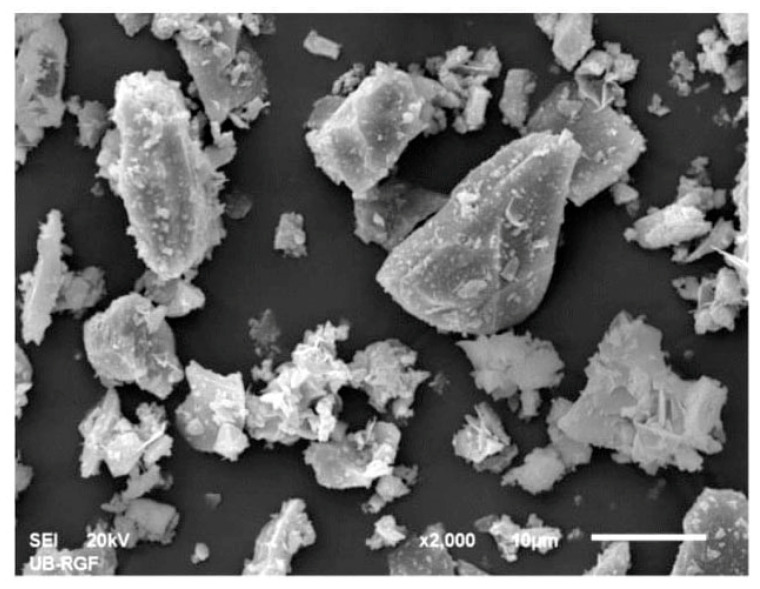
SEM microphotograph of synthesized cordierite filler.

The SEM microphotograph of the synthetized filler is given in Figure 2. From the microphotograph, it can be seen that big hexagonal angular grains are present in the observed grain mixture. These particles might correspond to the cordierite mineral phase, since cordierite is characterized by an orthorhombic pseudohexagonal symmetry, which gives way to a hexagonal symmetry at temperatures near the melting point [40]. Also, the cordierite particles are predominantly from 10 µm to 20 µm in size, which is in the same dimensional range as the biggest particles in the observed mixture. Quartz grains are usually classified as very angular [35]. Their diameter is usually around 15 µm [41]. Extremely angular particles of the previously stated average diameter are visible in the observed grain mixture. Corundum has a cubic crystal morphology [42]. The size of corundum particles is below 10 µm. The small cubically shaped grains are present in the filler mixture. The spinel mineral is characterized by sharp octahedral crystals [43]. The small needle-like or sharp octahedral particles are present in the observed grain mixture (periclase).

**Figure 3:**
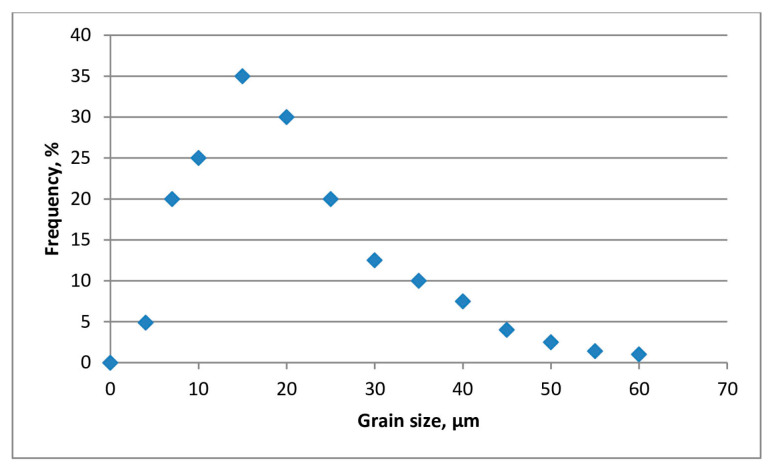
Grain size distribution of the synthetized cordierite filler.

**Figure 4:**
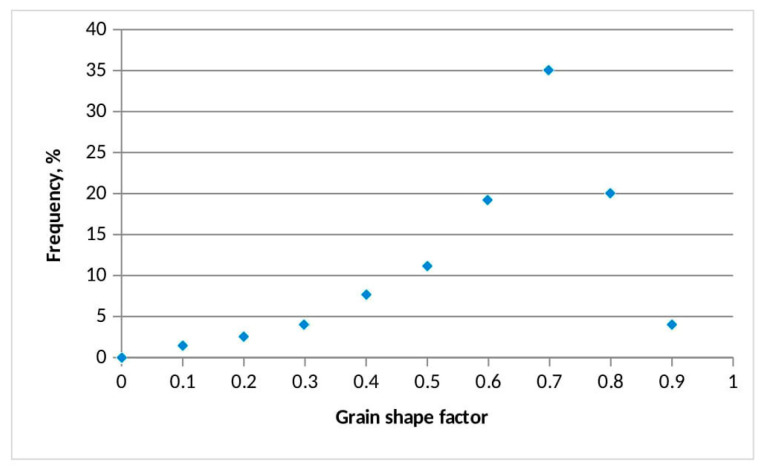
Grain shape factor distribution of the synthetized cordierite filler.

The measurement of the grain size and shape factor was performed on 3610 grains. The average grain size is 17.16 µm. The smallest and largest measured grain sizes are 1.04 and 57.16 µm, respectively. The standard deviation is σ = 10.46 µm. The average grain shape factor is 0.67. Based on the given data, the grains of this sample belong to the category of rounded grains. The maximum grain shape factor is 0.81 and the minimum grain shape factor is 0.15. The standard deviation is σ = 0.09. Figure 3 and Figure 4 show the histograms of the grain size and shape factor of the cordierite samples.

The prevailing grain shape factors (numeric values are adopted from the literature [30,42,44]) are in the range of 0.6 for roundness and 0.8 for sphericity (Grain shapes: http://grippo.pazsaz.com/hg252lab2.html, (accessed on 15 May 2024)). This means that the analyzed grains are not perfectly round or spherical (e.g., a perfect sphere has a shape factor that is equal to 1). The average grain is pseudo-round but with slightly sharp edges (grains sizing from 20 µm to 30 µm in the SEM microphotograph). The smaller grains (20–10 µm) are predominantly elongated, angular, and thin (grain shape factor 0.3 for both roundness and sphericity). The grains bigger than 30 µm have a 0.8 factor for roundness and sphericity. Namely, it can be said that the average grain in the observed mixture is sub-angular to sub-rounded, which makes this inorganic filler suitable for homogenous coating suspensions. The diversity in the particle size is useful because the particles with varying granulations help to create a uniform, continuous coating layer when applied to a solid surface.

### 3.2. Properties of Protective Coating: Superficial Damage Formation and Development

During the preparation and application of the coating on the metal substrate, no delamination was observed. When applied to an aluminum surface, the coating adheres readily and covers the surface well. The fluid does not leak or form lumps, bubbles, or drops due to uncontrolled flow. The coating dries easily in the air, does not break the dry layers, and does not rub.

**Figure 5:**
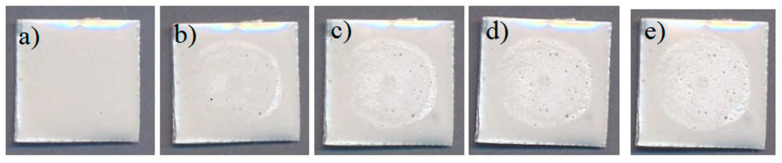
Photographs of the coated surface after cavitation erosion for the following durations: (**a**) 0; (**b**) 15; (**c**) 30; (**d**) 45; and (**e**) 60 min.

Changes in the surface of the coated sample (Figure 5) were explored during the analysis of resistance to cavitation using the ultrasonic vibration method. The mechanism of damage caused by cavitation was determined. Figure 5 shows the pictures of coated metal surfaces. Figure 5a–e demonstrate the evolution of the cavitation effect on the protective coating. Figure 5a illustrates the starting sample prior to cavitation erosion. The period of no mass loss, known as the incubation period, is brief, lasting approximately 2 min. After 15 min (Figure 5b), mild damage occurs at the peripheral edges of the sample. A small number of shallow pits emerge. After 30 min of cavitation erosion (Figure 5c), the additional pits emerge around the peripheral part of the sample exposed to the water stream. After 45 min (Figure 5d), the number of cavitation pits significantly increase, especially in the central part of the exposed coating (i.e., coated metal substrate). A total of 60 min of cavitation (Figure 5e) did not destroy or extremely deteriorate the coating; it produces a small number of deep pits. No cracks or delamination of the coating from the metal substrate are observed.

**Figure 6:**
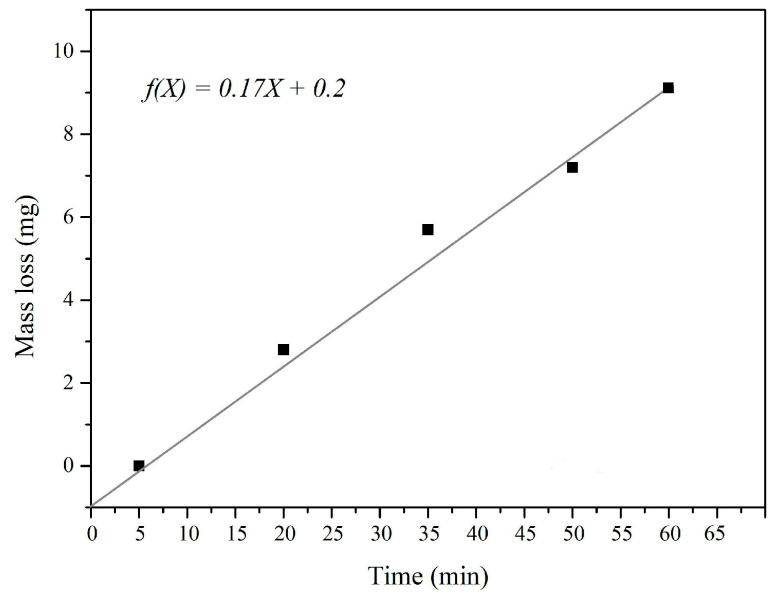
Cavitation erosion rate of the protective coating based on cordierite.

The formation of superficial pits, i.e., damage that occurs to the surface of the coating, is a result of the slow loss of mass from the coating’s surface. The mass loss depicts the damage outcomes after each cavitation sequence (i.e., 0, 15, 30, 45, and 60 min). After 15 min of erosion, the sample lost 3 mg, which is approximately 0.9% of the total sample weight. A total of 30 and 45 min of erosion caused 1.4% and 2.3% of mass loss, respectively. The highest mass loss (3.6%) was registered after 60 min of cavitation erosion. The rate of cavitation is calculated from the measured mass loss for every testing period. The points of the diagram are approximated by a straight line using the least squares method. The tangent of the slope depicts the loss of mass during the period of cavitation activity and represents the rate of cavitation erosion. For each set of the tested samples, three samples were used, and the findings represent the mean value of these measurements for each test interval. The cavitation rate given in Figure 6 is a quantifiable measurement of the intensity of material degradation caused by cavitation.

The calculated cavitation rate for the synthetized coating based on cordierite is 0.16 mg/min (as seen from the diagram provided in Figure 6). According to the photographs of the coated metal plates (Figure 5) and the mass loss measured during testing, it can be established that small pits arise in the early stages of the cavitation process, and their proportions fluctuate as the process progresses. The mass loss reflects and follows the pit creation process. It is observed that the cavitation erosion of the examined protective coating is a slow-paced process that shows the absence of cracks and no greater loss of coating mass. It can be assumed that there is also no loss of the coating’s protective characteristics, since the cavitation erosion runs slowly.

Cavitation erosion is a surface deterioration phenomenon that can cause significant damage to hydraulic devices and structural elements. As previously stated in the Introduction, the refractory coatings applied to the surfaces of hydraulic system equipment parts or underwater construction elements help to improve the base material’s resistance to wear and damage caused by cavitation. In order to reduce the speed with which damage to the coated surface is formed and progresses, special attention is paid to the preparation of the components and the production of the protective coating. The filler should be of high strength and hardness, and resistant to corrosion in order to achieve the surface protection of the substrate during the cavitation effect. The test results for various materials can be compared, since the ASTM G32 standard points to the fact that the tests conducted under the influence of cavitation must be conducted under identical test settings. This is crucial for the selection of materials to be used in particular exploitation conditions. The cavitation resistance of various materials can be assessed based on the value of the cavitation rate, which is defined by the ASTM G32 standard as the mass loss during the cavitation time. This is crucial for the selection of materials under particular exploitation conditions.

**Figure 7:**
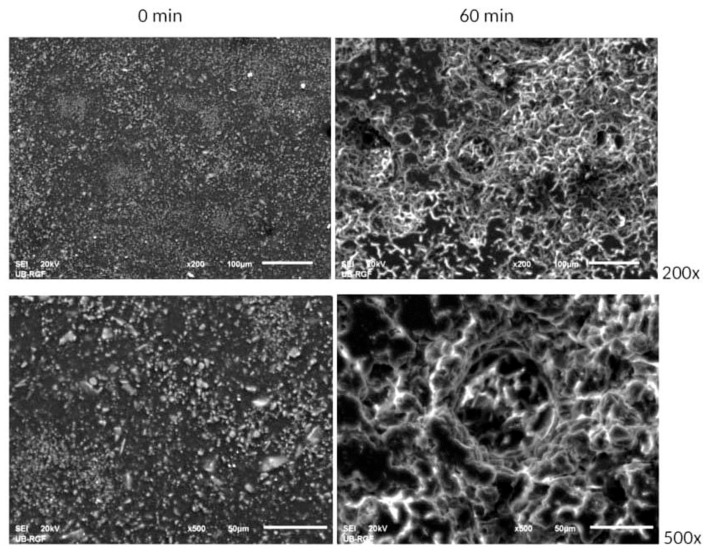
SEM microphotographs of the surfaces of the tested coating based on cordierite, before and after 60 min of exposure.

The SEM microphotographs (recorded with different optical zooms, shown in the picture) of the protective coating prior to cavitation erosion testing (i.e., 0 min) and after 60 min of exposure to water erosion are presented in Figure 7.

The original undamaged coating presented on the SEM microphotographs on the left depicts evenly distributed filler particles of different shapes and sizes immersed in the epoxy matrix. There are no damages, bubbles, or delamination of the coating applied to the metal substrate. The coating firmly adheres to the substrate, and during the exposure, no damage was observed, nor was there any loss of coating layers from the substrate. A good adhesion of the coating to the substrate was achieved; therefore, a good protection of the substrate was obtained under the effect of cavitation. The samples of the protective coating recorded after the final cavitation erosion period of 60 min are given on the right. As can be seen from the microphotographs, the pits are predominantly superficial. There are no void clusters of deeper channels starting from the superficial cavitation pits. The surfaces of the pits are smooth and seemingly shallow. Thereby, the metal substrate could not be damaged by the water stream action due to the presence of the protective coating.

The coating is a continuous layer on the substrate because the filler particles and the binder (epoxy matrix) are connected. This is found prior to the onset of cavitation, i.e., this can be seen in the SEM microphotographs of the samples recorded prior to the cavitation erosion—Figure 7, 0 min. Both the epoxy matrix and the filler particles are being eroded together at the same time during erosion. Following a 60 min exposure period, the protective layer’s surface sustained damage. The extensive observation revealed that whole-grain tearing, devoid of coating layer plastic deformation, is the primary mechanism of damage. As a result, the preparation of refractory fillers and their pulverization through the operations of grinding and micronization to the desired sizes and shapes of powder grains received particular attention during the planning of the sample preparation. The grain crushing helps reduce the rate of damage to the coating layers, and the applied refractory fillers are highly resilient to cavitation erosion. After 60 min of exposure, the coating developed smooth, acute dimples on its surface. This suggests that the coating mass gradually decreased without the plastic deformation of the material.

## 4. Conclusions

In this study, protective coatings based on cordierite filler (88 wt.%) and epoxy resin (7 wt.%) for metal substrates (aluminum) were produced and tested for cavitation erosion resistance. Based on the research results, the following conclusions can be drawn:

- To acquire protective coatings with pre-designed properties, extra attention should be devoted to component selection from the coating composition, as well as to studies of coating production techniques. Grinding and mechanical activation processes are used to produce refractory fillers with certain grain sizes and shapes. For the synthesis of coatings with good rheological properties, the protection of metal and non-metal surfaces, and the application in harsh exploitation conditions (effect of wear, corrosion, and cavitation), to which parts of equipment in the process industry are exposed, the choice of binders, organic solvents, and additives to maintain the suspension is crucial. A high-strength, corrosion-resistant filler with high hardness and strength is chosen beforehand to guarantee the substrate’s surface protection over time. To minimize the rate at which damage to the coated surface forms and progresses, extra care is taken in filler preparation.
- The average grain in the filler mixture is sub-angular to sub-rounded, making it suitable for homogeneous coating solutions.
- The protective coating adhered properly to the aluminum substrate, completely covering the surface and leaving no bumps or bubbles. The coating dries quickly in the air and exhibits no delamination.
- The cavitation rate of 0.16 mg/min suggests a slow deterioration of the coating. The morphology of the protective coating samples from the last cavitation erosion phase of 60 min revealed the predominantly superficial pits. There are no empty clusters of deeper channels beginning with the surface cavitation pits. Extensive observation has revealed that whole-grain tearing, without the plastic deformation of the coating layer, is the primary damage mechanism. The presence of the protective layer prevented the metal substrate from being harmed by water stream action. This study shows that the proposed protective coating may be used in environments with cavitation loads and provides protection for the substrate material, which will be tested on a semi-industrial scale in the next step of the study.

## Data Availability

The original contributions presented in the study are included in the article, further inquiries can be directed to the corresponding author.
